# Composite-derived monomers affect cell viability and cytokine expression in human leukocytes stimulated with *Porphyromonas gingivalis*

**DOI:** 10.1590/1678-7757-2018-0529

**Published:** 2019-06-03

**Authors:** Sheyla Omonte Neves, Luísa Mourão Dias Magalhães, Jôice Dias Corrêa, Walderez Ornelas Dutra, Kenneth John Gollob, Tarcília Aparecida Silva, Martinho Campolina Rebello Horta, Paulo Eduardo Alencar Souza

**Affiliations:** 1 Pontifícia Universidade Católica de Minas Gerais, Departamento de Odontologia, Programa de Pós-graduação em Odontologia, Belo Horizonte, Minas Gerais, Brasil.; 2 Universidade Federal de Minas Gerais, Instituto de Ciências Biológicas, Departmento de Morfologia, Belo Horizonte, Minas Gerais, Brasil.; 3 International Research Center, A.C.Camargo Cancer Center, São Paulo, SP, Brasil.; 4 Universidade Federal de Minas Gerais, Faculdade de Odontologia, Belo Horizonte, Minas Gerais, Brasil.; 5 Instituto Nacional de Ciência e Tecnologia em Doenças Tropicais – INCT-DT, Belo Horizonte, Minas Gerais, Brasil.

**Keywords:** Cytokines, Porphyromonas gingivalis, Mononuclear leukocytes, Materials testing, Composite resins

## Abstract

**Objectives:**

Dental composites release unreacted resin monomers into the oral environment, even after polymerization. Periodontal cells are, therefore, exposed to substances that potentially elicit the immune inflammatory response. The underlying molecular mechanisms associated with the interaction between resin monomers and human immune cells found in the gingival crevicular fluid are not fully understood yet. This study investigated the ability of bisphenol A-glycidyl methacrylate (BISGMA), urethane dimethacrylate (UDMA) and triethylene glycol dimethacrylate (TEGDMA) to induce apoptosis and cytokine release by human leukocytes stimulated with a periodontal pathogen.

**Methodology:**

Peripheral blood mononuclear cells (PBMC) from 16 healthy individuals were included in this study. To determine the toxicity, the PBMC were incubated for 20 hours, with monomers, for the analysis of cell viability using MTT assay. To evaluate cell death in the populations of monocytes and lymphocytes, they were exposed to sub-lethal doses of each monomer and of heat-inactivated *Porphyromonas gingivalis* (*P. gingivalis*) for 5 hours. Secretions of IL-1β, IL-6, IL-10 and TNF-α were determined by ELISA after 20 hours.

**Results:**

UDMA and TEGDMA induced apoptosis after a short-time exposure. Bacterial challenge induced significant production of IL-1β and TNF-α (p<0.05). TEGDMA reduced the bacterial-induced release of IL-1β and TNF-α, whereas UDMA reduced IL-1β release (p<0.05). These monomers did not affect IL-10 and IL-6 secretion. BISGMA did not significantly interfere in cytokine release.

**Conclusions:**

These results show that resin monomers are toxic to PBMC in a dose-dependent manner, and may influence the local immune inflammatory response and tissue damage mechanisms via regulation of bacterial-induced IL-1β and TNF-α secretion by PBMC.

## Introduction

The employment of resin-based materials in Dentistry is presently ubiquitous. Dental composite resins comprise a complex mixture of materials, usually consisting of an organic matrix, reinforcing inorganic filler and a silane-coupling agent that connects the filler and resin matrix.^1^ Currently, the most common resin systems used in dentistry are methacrylates.^1^ In these systems, the polymerizable organic matrix contains one or more base monomers, such as bisphenol A-glycidyl methacrylate (BISGMA), urethane dimethacrylate (UDMA), and diluent monomers like triethylene glycol dimethacrylate (TEGDMA).^2^

Methacrylate monomers are key components that help achieving the required characteristics of resin-based dental materials. Stimuli such as light induce the transformation of monomers into polymers; however, the complete reaction is still a challenge. The literature suggests the best polymerization one can achieve in freshly light-cured dental composites is around 30-40%, which might increase up to 50-70% after 24 h.^3^ As a consequence of this relatively poor polymerization, unreacted resin monomers may be released from dental composites, particularly in the first 24 hours after placement.^4^ ^,^ ^5^ Then, monomers elute for many days after polymerization, both due to incomplete curing and as a consequence of natural degradation.^4^ This fact undermines biocompatibility, and there is evidence that monomers act by disturbing the environment, ultimately influencing the function of various cell types.^6^ ^–^ ^8^

In this regard, adverse biological reactions to resin components, such as local and systemic toxicity, pulp reactions, allergy, genotoxicity and cytotoxicity, have been reported.^6^ Nonetheless, studies targeting PBMC-derived immune cells stimulated with periodontal pathogens, commonly found in the gingival sulcus, are scarce. In addition, the underlying mechanism associated to the effect of sub-lethal doses of resin monomers in periodontal inflammation remains to be elucidated.

The presence of dental biofilms keeps a persistent sub-clinical inflammatory infiltrate in the gum tissue.^9^ ^,^ ^10^ *Porphyromonas gingivalis* (*P. gingivalis*), a Gram-negative anaerobic bacteria that colonizes sub-gingival tissue sites, is an important pathogen associated with chronic periodontitis.^11^ The immune system recognizes pathogenic invaders such as *P. gingivalis* and initiates an inflammatory response, launching the production of numerous inflammatory mediators such as pro- and anti-inflammatory cytokines. IL-1β, TNF-α and IL-6 are pro-inflammatory cytokines that enhance bone resorption and activate lymphocytes,^12^ whereas IL-10 displays anti-inflammatory properties and inhibits bone resorption.^13^ These molecules are regulated by tightly controlled pathways and orchestrate the inter-cellular communication, coordinating host immune response.^9^ Mononuclear leukocytes play a critical role in this process.^9^

Although the bacterial biofilm is important in periodontitis initiation and progression, it is primarily the host's inflammatory response that promotes considerable damage to periodontal tissues.^11^ In fact, a delicate balance between health and disease maintains tissue integrity.^4^ Any disability or excess of inflammatory response may result in tissue damage. In this scenario, the contact of a composite resin restoration with gingival tissues, and the local release of monomers, may cause changes in periodontal cellular processes, acting as a local stressor.

In this study, we focused on evaluating the effects of resin monomers on leukocytes stimulated or not by *P. gingivalis*. To the best of our knowledge, this is the first report on the effect of monomers exposure in leukocytes stimulated with a periodontal pathogen.

## Methodology

### Chemicals

Three monomers (BISGMA, TEGDMA and UDMA) were chosen, as they are constituents of a variety of dental resin systems. All monomers were obtained from Sigma-Aldrich, St. Louis, MO, USA, and dissolved in Dimethyl sulfoxide (DMSO; Sigma-Aldrich, St. Louis, MO, USA).

### Peripheral blood mononuclear cells (PBMC) preparation

The study protocol was approved by the Research Ethics Committee of the Pontifical Catholic University of Minas Gerais, Minas Gerais, Brazil.

Sixteen healthy individuals were recruited. All of them gave informal consent to participate. Inclusion criteria were: individuals between 25 and 50 years old, who did not report any systemic or oral disease, were not immunocompromised and did not use drugs that could affect the blood, the immune system or antimicrobial medication.

Blood samples (10 mL) were drawn by peripheral venipuncture into heparinized vacutainer tubes (Becton and Dickinson, Franklin Lakes, NJ, USA). Purification of mononuclear cells was performed as described previously.^5^ Briefly, heparinized blood was diluted to a proportion of 1:1 with phosphate-buffered saline (PBS) (Sigma Aldrich, St. Louis, MO, USA). PBMC (peripheral blood mononuclear cells) were isolated from peripheral blood by density gradient centrifugation in Ficoll-Paque (GE Healthcare Bio-Sciences, Uppsala, Sweden). The PBMC interface was collected and washed three times by centrifugation with phosphate-buffered saline (PBS) and re-suspended in RPMI 1640 (Sigma Aldrich, St. Louis, MO, USA), then supplemented with antibiotic-antimycotic [0.25 g/mL amphotericin B, 200 U/mL penicillin, and 0.1 mg/mL streptomycin (Sigma, St. Louis, MO, USA)] and L-glutamine [1 mM (Sigma, St. Louis, MO, USA)].

### Determination of the monomers toxicity curve (TC)

The PBMC from 8 individuals were seeded in a 96-well plate (2.5×10^5^ cells/well) and incubated with serial dilutions (2-fold dilutions) of BISGMA (1,000 to 7.8 μM), TEGDMA (10,000 to 78 μM) and UDMA (2,000 to 15 μM) for 20 h at 37°C in a 5% CO_2_ atmosphere. Monomer concentrations were based on previous studies.^8^ ^,^ ^14^ ^–^ ^16^ The resin monomers stock solutions were diluted down to obtain an up to 0.9% (v/v) final concentration of DMSO in cell cultures. Cells were then subjected to a colorimetric functional assay (MTT) for assessing mitochondrial activity. Experiments were performed in triplicate. Positive control groups were composed of cells in complete RPMI with 0.9% DMSO and cells without DMSO. Negative control was composed of cells heated at 100°C for 15 min. After 20 h of incubation, plates were centrifuged (1200 rpm, 22°C, 10 min). The culture medium was removed from each well, and the cells re-suspended in 110 μL of methyl tetrazolium (MTT, 2.5 mg/mL in RPMI-1640 solution 10% v/v) and incubated for 4 h at 37°C in a 5% CO_2_ atmosphere. The viable cells metabolized the yellow MTT and generated the purple formazan product, which was dissolved in 100 μL of DMSO and read against blank reagent at 575 nm using a micro plate reader (Molecular Devices, Sunnyvale, CA, USA). The concentration of each monomer that caused a 50% (TC_50_) and 20% (TC_20_) decrease in the cell metabolic activity was interpolated from the dose-response curves. This was carried out by fitting the experimental data to a sigmoidal curve using the least mean square algorithm.

### Bacterial preparation

A single strain of *Porphyromonas gingivalis* ATCC 33277 was purchased from ATCC (ATCC, Manassas, VA, USA). *Porphyromonas gingivalis* were cultured in Brucella agar (Becton, Dickinson and Company, Sparks, MD, USA) supplemented with 5% blood, 0.5% yeast extract, 1% of hemin (Sigma Chemical Co., St. Louis, MO, USA) and 0.1% menadione (Sigma Chemical Co., St. Louis, MO, USA) under anaerobic conditions (Bactron Anaerobic Chamber) (Sheldon Manufacturing Inc., Cornelius, OR, USA) with an atmosphere of 85% N_2_, 5% CO_2_, and 10% H_2_, at 37°C for 72 h. To quantify CFU, the bacteria were cultivated in Brucella Broth supplemented (BBS) with 5% blood, 0.5% yeast extract, 1% of hemin and 0.1% menadione in the same conditions. After 48 h, serial dilutions were performed from 10^−1^ to 10^−8^ and 0.1 mL was seeded on BBS, in triplicate, to count the CFU. To confirm the concentration of bacteria in BBS after a 48-h growing, a spectrophotometer (Utrospec 10 Cell Density Meter) (Biochrom, Cambridge, UK) was used at OD 650 wavelength. Bacterial cultures were then washed in PBS and heat inactivated at 100°C for 30 min, as described previously by other authors.^17^ The stock solution was kept in a −80°C freezer, thawed and diluted prior to experiments. The appropriate dilutions were made to achieve the desired concentration (MOI 100:1).

### Evaluation of necrosis and apoptosis

The APC Annexin V apoptosis detection kit with 7AAD (Biolegend, San Diego, CA, USA) was used to measure apoptosis and necrosis. Annexin V has affinity to phosphatidylserine, a phospholipid translocated to the cell extracellular membrane in the earlier stages of apoptosis. To distinguish early apoptotic cells from late apoptotic/necrotic cells, the vital dye 7AAD was used.

**Figure 1:**
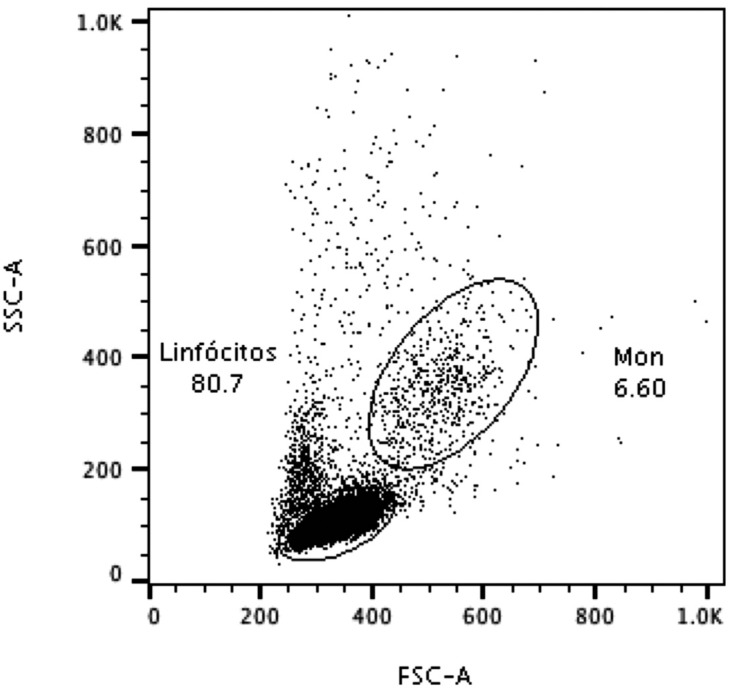
Representative lymphocyte and monocyte populations gated in the size *versus* granularity dot plot graph

**Figure 2:**
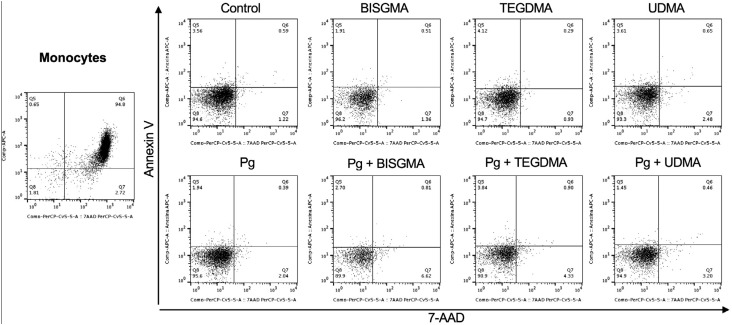
Dot plot graphs representative of Annexin V and 7AAD staining in the monocyte population after exposure to resin monomers and *P. gingivalis*. The dot plot on the left shows the monocyte population in the positive control (heat-killed peripheral blood mononuclear cells)

**Figure 3:**
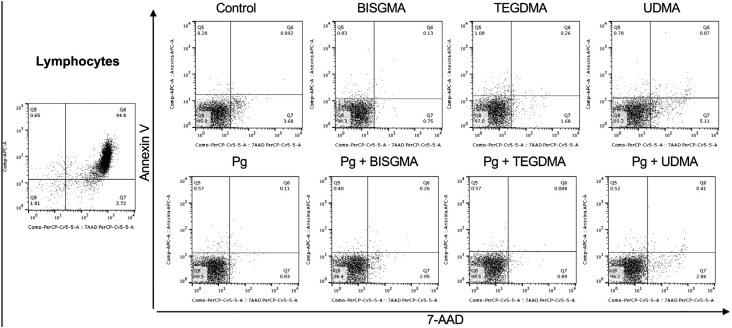
Dot plot graphs representative of Annexin V and 7AAD staining in the lymphocyte population after exposure to resin monomers and *P. gingivalis*. The dot plot on the left shows the lymphocyte population in the positive control (heat-killed peripheral blood mononuclear cells)

Cells from 8 individuals were re-suspended in complete RPMI (RPMI supplemented with antibiotic-antimycotic and L-glutamine) to a concentration of 1.0×10^6^ cells/mL. Cells were then subjected to eight conditions: 1) Control (media), 2) BISGMA, 3) TEGDMA, 4) UDMA, 5) *P. gingivalis*, 6) *P. gingivalis* + BISGMA, 7) *P. gingivalis* + TEGDMA, 8) *P. gingivalis* + UDMA. In the groups stimulated with *P. gingivalis*, cells were challenged with heat-inactivated *P. gingivalis* at moi: 100 (1.0×10^8^ CFU/mL). All samples were cultured for 1 h, at 37°C in an atmosphere with 5% CO_2_. Next, resin monomers were added to the test group samples to a final concentration of their TC_20_. Cells were incubated for additional 4 h at 37°C, 5% CO_2_, totaling an incubation time of 5 h. Then, cells were washed twice with cool Cell Staining Buffer (Biolegend, San Diego, CA, USA) and collected by centrifugation. Cell cultures were re-suspended in 0.1 mL Annexin V Binding Buffer containing Annexin V-APC and 7AAD, and incubated in the dark for 15 min. The cell cultures were then centrifuged and re-suspended in 0.15 mL PBS. Samples were acquired on a FACSCanto™ II (Becton Dickinson, San Jose, CA, USA) flow cytometer and analyzed using FlowJo version 7.6.5 (Tree Star Inc, Ashland, OR, USA). Monocytes and lymphocytes populations were selected according to their location in the graph of size vs granularity (Figure 1). Results are expressed as the percentage of stained cells for a given marker (Annexin V and 7AAD) in the monocyte (Figure 2) and lymphocyte gate (Figure 3). Viable (Annexin V^−^; 7AAD^−^) cells were counted in the lower left quadrant of density plots, and the percentage of apoptotic cells (Annexin V^+^; 7AAD^−^) and late-apoptotic/necrotic cells (Annexin V^+^; 7AAD^+^) were determined accordingly.

### Measurement of inflammatory cytokines

To analyze the production of cytokines, PBMC (0.5×10^6^ cells/well) from 16 healthy individuals (9 females, 7 males) were seeded in 24-well plates and incubated under the above-mentioned conditions. Samples were cultured for 1 h, at 37°C in an atmosphere with 5% CO_2_. The resin monomers were then added to the test group samples to a final concentration of their TC_20_. Cells were incubated for additional 19 h at 37°C, 5% CO_2_, making a total incubation time of 20 h. Next, we performed an experiment to analyze the influence of TEGDMA on the PBMC from 8 individuals (5 females, 3 males) after 5 h of incubation. Cells were incubated with *P. gingivalis* for 1 h, followed by the addition of TC_20_ TEGDMA, and then re-incubated for 4 h. All samples were centrifuged (3000 rpm, 4°C, 6 min) and the supernatant was collected for analysis. The production of IL-1β, IL-6, TNF-α and IL-10 was determined by double-ligand enzyme-linked immunosorbent assay (ELISA), according to the manufacturer's protocol (R&D systems, Minneapolis, MN, USA). Results were expressed as picograms of cytokine/mL.

### Statistical analysis

The Shapiro-Wilk test showed whether data came from a normal distribution. All samples were submitted to a rout test to identify outliers. Data that met the criteria for parametric tests were analyzed by a Student's paired t-test or repeated-measurements one-way ANOVA, followed by Tukey's test. Data groups that failed normality tests were analyzed by Wilcoxon or Friedman test, followed by Dunn's test. Correlations between cytokine levels and cell death data were calculated using Spearman correlation coefficients.

The level of significance was set at 5%. These analyses were performed using the software GraphPad Prism 5:01 (GraphPad Software, San Diego, CA, USA).

## Results

### Analysis of PBMC viability/metabolic (mitochondrial) activity

**Figure 4:**
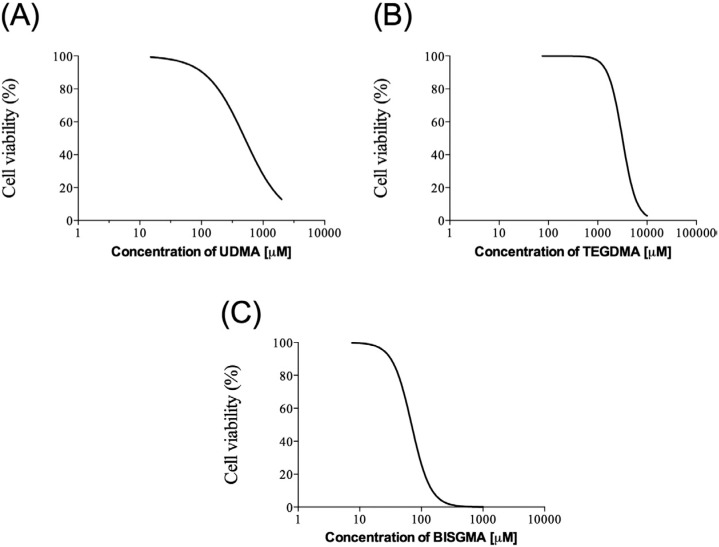
Dose-response curves for peripheral blood mononuclear cells (PBMC) treated with monomers, after 20 hours of exposure. Cell viability was assessed by a MTT-based assay and expressed as a percentage of control (media). Curves correspond to a sigmoidal fitting. Data from 8 donors are shown. All experiments were performed in triplicate

The MTT assay revealed that all substances tested induced a dose-dependent cytotoxicity on PBMC (Figure 4). Positive control groups, both with and without DMSO, showed similar results for cell mitochondrial activity. As expected, negative control cells (heated cells) presented low absorbance (0-7%). In high concentrations, monomers exhibited toxic effects on PBMC and inhibited the MTT cell metabolization. UDMA at concentrations of 0.05 – 2 mM decreased the mitochondrial activity of PBMC by 50-93% (Figure 4A). TEGDMA at concentrations of 2.5 – 10 mM diminished cell activity by 26-93% (Figure 4B), whereas BISGMA at 0.06 – 1 mM caused a 44-95% decrease (Figure 4C). Therefore, the following order of toxicity (TC_50_ values) was observed (μM): BISGMA 69.0 (62.1-77.3, 98% confidence interval), UDMA 505.0 (429.1-593.9, 95% confidence interval) and TEGDMA 3161.0 (2697.0-3708.0, 90% confidence interval), and the TC_20_ values were 50.5 μM, 167.0 μM and 2150.0 μM for BISGMA, UDMA and TEGDMA, respectively. The TC_20_ values were used in the following experiments.

### Evaluation of apoptosis and necrosis using flow cytometry

**Figure 5:**
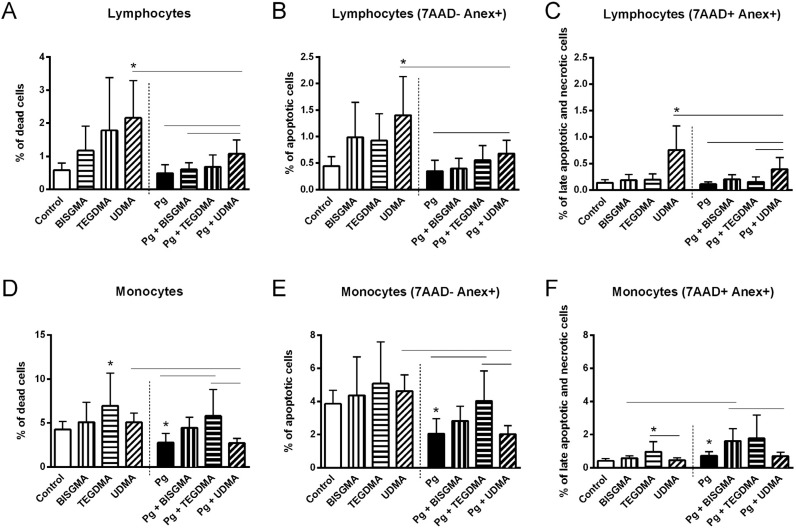
Induction of cell death, apoptosis and necrosis in peripheral blood mononuclear cells (PBMC) after 5 hours. Cell cultures were exposed to TC^20^ TEGDMA, UDMA, BISGMA, *P. gingivalis* or a combination of *P. gingivalis* and each resin monomer at their TC^20^ concentration. Results are expressed as average values for percentage of cells. Error bars indicate the standard deviation. Data from 8 donors are shown * represents a significant difference (p<0.05) regarding the control group, and connecting lines represent significant differences (p<0.05) between the experimental groups

To check the influence of the different monomers in cell death, as well as for determining the occurrence of apoptosis and necrosis, cells were exposed to heat-inactivated *P. gingivalis* or left undisturbed for 1 h, incubated with TC_20_ BISGMA, TEGDMA or UDMA for additional 4 h and stained with Annexin V and 7AAD for flow cytometric analysis. Figure 5 shows the percentage of dead cells, as well as the percentage of apoptotic and necrotic lymphocytes and monocytes after treatment with each of the monomers, either previously stimulated or not with *P. gingivalis*. Evaluation of the % Annexin V^+^ and % 7AAD^+^ cells demonstrated that heat-inactivated bacteria (*P. gingivalis* group) did not influence the frequency of dead cells when compared with media control (Figure 5A and 5D), although a decrease in the number of apoptotic monocytes was observed after incubation with *P. gingivalis*, in comparison to control (Figure 5E). Then, we evaluated the effects of exposure to resin monomers. The incubation with BISGMA and *P.gingivalis* induced a significant increase in the percentage of necrotic monocytes compared with BISGMA alone (Figure 5F). Apart from that, no other changes were observed in lymphocytes and monocytes death following incubation with this monomer, regardless of *P. gingivalis* stimulus. TEGDMA did not interfere with lymphocyte death (Figure 5A). On the other hand, TEGDMA incubation produced a statistically significant increase in monocyte death when compared with media control (Figure 5D). Likewise, TEGDMA associated to *P. gingivalis* produced a higher percentage of dead monocytes compared to *P. gingivalis* alone (Figure 5D). When analyzing the percent of apoptotic monocytes, one can observe an increased apoptosis of cells incubated with TEGDMA and *P. gingivalis* in comparison to cells incubated with *P. gingivalis* alone (Figure 5E), while no differences were observed in the frequency of necrotic cells, suggesting that most monocyte death was due to apoptosis. UDMA significantly increased lymphocyte death (Figure 5A), associated with increased frequency of both apoptotic and necrotic lymphocytes, despite bacterial stimulation (Figure 5B and 5C). Surprisingly, co-incubation with *P. gingivalis* and UDMA significantly reduced lymphocyte death by apoptosis and necrosis when compared with incubation with UDMA alone (Figure 5A, 5B and 5C). A similar fact was also observed in the monocyte population, where it reduced cell death by apoptosis, with no interference in necrosis (Figure 5D, 5E, 5F). It is worth noting that UDMA lead to greater lymphocyte death than TEGDMA or BISGMA, which had more effect on monocytes. From Figure 5, one can see that the UDMA-induced cell death mechanism, as well as the other monomers, was mainly apoptosis, as the frequency of apoptosis was higher than necrosis.

### Cytokine release analysis

**Figure 6:**
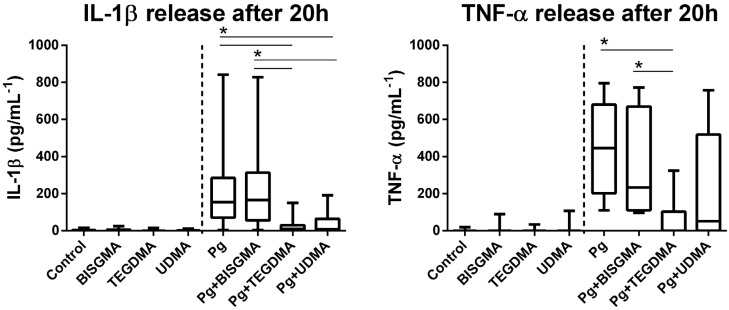
Cytokine release by PBMC, whether challenged or not with *P. gingivalis*, in the presence or absence of resin monomers at TC20 concentrations. Culture supernatants were harvested at 20 h and tested for the secretion of cytokines using ELISA analysis. Graph bars represent the median values with range. Data from 16 donors are shown * represents a significant difference (p<0.05) regarding the control group, and connecting lines represent significant differences (p<0.05) between the experimental groups

PBMC were exposed to heat-inactivated *P. gingivalis* or left untreated for 1 hour, and then incubated with TC_20_ BISGMA, TEGDMA or UDMA. After 20 hours, the resin monomers did not affect cytokine production when compared to the incubation with media alone (Control group) (Figure 6). *P.gingivalis* led to a significant increase in the production of the pro-inflammatory cytokines IL-1β and TNF-α by PBMC in comparison to the Control group (Figure 6).

**Figure 7:**
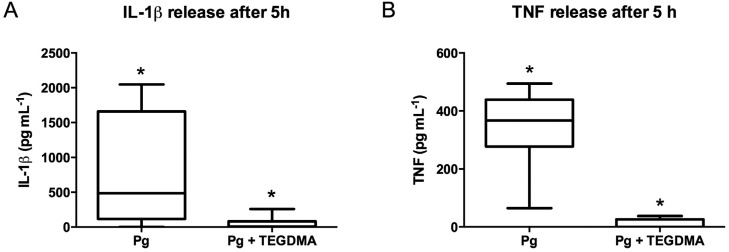
Effect of TEGDMA on bacterial-induced cytokine release by peripheral blood mononuclear cells (PBMC). Culture supernatants were harvested at 5 h and tested for the secretion of cytokines using ELISA analysis. Graph bars represent the median values with range. Data from 8 donors are shown * represents a significant difference (p<0.05) between the groups

Secretion of IL-1β and TNF-α was not altered in cells co-incubated with *P. gingivalis* and BISGMA after 20 h when compared to *P. gingivalis* stimulation alone (Figure 6). Interestingly, the TEGDMA treatment inhibited the production of pro-inflammatory cytokines IL-1β and TNF-aby cells challenged with *P. gingivalis* after 5 and 20 h of incubation in comparison to the *P. gingivalis* stimulation alone (Figures 6 and 7). Analysis of correlation between cytokine levels and cell death did not show any negative correlation that could link the great cell death to a decreased production of these cytokines. However, UDMA exposure statistically decreased the IL-1β secretion by PBMC stimulated with *P. gingivalis* (Figure 6A), and a negative correlation between IL-1β levels and the percentage of apoptotic lymphocytes was observed (rho= −0.9, p=0.002), indicating the decreased IL-1β production could be linked to a decreased number of cells. No influence was observed in TNF-α secretion (Figure 6B).

In all groups, in the absence of *P. gingivalis*, no detectable quantities of IL-10 were observed after 5 and 20 h. While IL-6 was detected in the supernatants from cultures in all groups, no statistical differences among groups was observed. The release of IL-10 and IL-6 was not influenced neither by the treatment with *P. gingivalis* nor by the monomers incubation (data not shown).

## Discussion

Although methacrylate monomers may be released from resinous materials and interact with immune cells, there is scarce data regarding the influence of these chemicals on functional characteristics of human PBMC-derived immune cells, i.e., mononuclear leukocytes. Here, we found that resin monomers may influence the local immune inflammatory response and tissue damage mechanisms via regulation of bacterial-induced IL-1β and TNF secretion by PBMC.

Regardless of the periodontium complexity and the limitations of *in vitro* conditions, we challenged mononuclear leukocytes with *P. gingivalis* before exposure to the resin monomers, aiming to mimic some conditions that might likely happen in the oral cavity. With such methodology, we observed the cytotoxicity of UDMA and TEGDMA and that, also, bacterial challenge induced a significant production of IL-1β and TNF. Interestingly, TEGDMA reduced bacterial-induced release of IL-1β and TNF, whereas UDMA reduced IL-1β release. Our experiments revealed a high dose-dependent cytotoxicity for BISGMA, UDMA and TEGDMA. This result is similar to previous studies that observed important cytotoxic effects associated to diverse cell lines incubated with resin monomers.^16^ ^,^ ^18^ It should be noted that some authors proved that the susceptibility and determination of TC_50_ to a monomer depend on the type of host cells.^15^ The PBMC TC_50_ to TEDGMA was 3161 μM. Using primary human gingival fibroblast cells, the TEGDMA TC_50_ value was 3460 μM.^16^ In contrast, human THP-1 monocytes displayed TC_50_ of 1500 μM.^18^ Furthermore, the PBMC TC_50_ to BISGMA was 69 μM, whereas other authors found 31 μM for immortal human endothelial cells.^14^ UDMA exhibited a TC_50_ value of 505 μM in this study, while 106 μM for primary human gingival fibroblasts was previously described.^16^ Therefore, our results confirmed that cytotoxicity varied considerably depending on the cell type and on the resin monomer tested. The TEGDMA TC_50_ was considerably higher than that observed for UDMA, and BISGMA presented the lowest value. This result is corroborated by other studies that observed a cytotoxicity ranking as follows: BISGMA>UDMA>TEGDMA.^6^ ^,^ ^16^ However, most articles on this subject report the use of TC_50_, and the authors of this study decided to use the TC_20_ because a 50% toxic concentration toxic in the cell population compromises too many cells and seems to be above the level one may expect to find in the periodontal environment exposed to resin composites.^4^

This research provides experimental evidence that UDMA induces apoptosis in lymphocytes, as described by other authors.^19^ It has been suggested this resin monomer induces apoptosis through DNA lesion or mitochondrial dysfunction.^19^ Heat-inactivated *P. gingivalis* did not induce apoptosis in lymphocytes after 5 hours of culture. This finding is similar to a previous study that tested PBMC stimulated with the same bacteria.^20^ Interestingly, co-incubation of UDMA and *P. gingivalis* led to a statistically significant decrease in the number of apoptotic lymphocytes when compared to UDMA alone. As apoptosis can be triggered by the imbalance between pro- and anti-apoptotic intracellular molecules, it is tempting to hypothesize that *P. gingivalis* may interfere with the signaling pathways associated with the expression of these molecules. In fact, previous studies demonstrated that activated lymphocytes express molecules that antagonize apoptosis.^21^ The pathway involved in the protective effect of heat-killed *P. gingivalis* on UDMA-exposed PBMC is an intriguing issue to be explored. It is worth mentioning that UDMA did not significantly affect monocytes viability, inducing lymphocyte death primarily via apoptosis.

After a short-time exposure to TEGDMA, we observed an increased monocyte death mainly via apoptosis. Such result is similar to that detected by other authors in human pulp-derived cells after 6 h of incubation.^22^ Additionally, we showed that BISGMA did not significantly affect cell death via apoptosis or necrosis in lymphocytes and monocytes. As a matter of fact, the frequency of annexing V-positive cells in our experimental groups was low. Some studies demonstrated that apoptosis caused by resin monomers is time- and dose-dependent.^22^ ^,^ ^23^ For instance, BISGMA exhibited a dose-dependent apoptotic effect in dental pulp cells after 24 hours of incubation.^23^ Thus, an experiment with longer exposure periods might render higher rates of apoptosis related to TEGDMA and UDMA exposure and may help determine whether sub-lethal doses of BISGMA induce apoptosis in human mononuclear leukocytes.

As demonstrated, TC_20_ BISGMA, TEGDMA and UDMA did not alter cytokine production by unstimulated human mononuclear leukocytes. These findings are similar to data obtained using fibroblasts and THP-1 macrophages.^8^ ^,^ ^15^ This speaks in benefit of likely favorable properties of resin monomers in low concentrations, which would be an important assurance when using resin composites in dental treatments.

After co-stimulation with bacteria, the methacrylate monomer UDMA decreased IL-1β release by PBMC. Moreover, TEGDMA inhibited the secretion of both IL-1β and TNF-α. Previous studies using murine or immortalized cells described similar results.^7^ ^,^ ^8^ ^,^ ^18^ ^,^ ^22^ However, it should be pointed out the lower secretion of cytokines could be due to a decrease on cells able to produce then. In fact, we observed a negative correlation between IL-1β levels and the percentage of apoptotic lymphocytes incubated with UDMA. However, we did not find similar correlation between levels of TNF-α or IL-1β and cells incubated with TEGDMA, suggesting that other mechanisms could be linked to the decreased production of cytokines by PBMC cells.

Our experimental conditions were closer to the actual conditions in the gingival crevice, when compared to previous studies.^7^ ^,^ ^8^ First, we analyzed fresh human PBMC, blood cells prone to inflammatory cell recruitment, whereas other studies tested human and mouse immortalized cell lines.^7^ ^,^ ^8^ Furthermore, we tested monomers exclusively in their TC_20_ concentration, a sub-lethal dose, as the literature suggests that large quantities of monomers are not expected to be released from dental fillings.^4^ ^,^ ^24^ Lastly, we used heat-inactivated *P. gingivalis*, a major periodontal pathogen,^13^ whereas other studies used LPS from *E. coli*. As different bacteria act differently in the same cell, triggering different signal pathways,^25^ a study such as this one - analyzing bacteria with oral relevance - is important to verify their possible effects in the periodontal tissue.

IL-1β and TNF are pro-inflammatory cytokines that regulate host response.^12^ In particular, IL-1β is a cytokine whose bioactivity is controlled by activation of inflammasome complexes. Recent data demonstrated that LPS exposure alone is capable of activating inflammasomes in human monocytes, key cells of the host's early response to bacterial stimuli.^26^ Accordingly, these cytokines have been implicated in the pathogenesis of tissue destruction in periodontitis.^12^ In our study, *P. gingivalis* was responsible for a significant increase on the cytokine expression by leukocytes, and TEGDMA and UDMA exposure reduced the secretion of these molecules. Similarly, a study performed in human THP-1 macrophages demonstrated that dental resin monomer-based materials inhibited LPS-induced IL-1β and TNF secretion at concentrations that suppressed mitochondrial activity by 50%, TC_50_.^27^ The present results were obtained at TC_20_ concentrations; hence, a lower concentration of resin monomers is still capable of alter normal leukocyte-directed inflammatory responses. This is a relevant finding since the first step in examining material biocompatibility is to analyze its effect on basic cell function. As components such as co-stimulatory molecules and other cytokines are involved in the immune response, further studies are important to fully delineate the relationship among resin monomers, inflammation, host response, and periodontal tissue health and disease.

## Conclusion

The effect of resin monomers on human leukocytes stimulated with a periodontal pathogen, shown for the first time in this paper, indicates that sub-lethal doses of TEGDMA and UDMA induce cell death via apoptosis and/or necrosis depending on the cell type, in addition to influencing PBMC cytokine production. Our results suggest that resin monomers interfere with the local immune inflammatory response and with the tissue damage mechanisms associated with IL-1β and TNF secretion.
